# How Do Fear-Avoidance and Catastrophizing Pain Beliefs Affect Functional Status and Disease Activity in Axial Spondyloarthritis?

**DOI:** 10.3390/medicina61061039

**Published:** 2025-06-05

**Authors:** Carlos Fernández-Morales, María de los Ángeles Cardero-Durán, Manuel Albornoz-Cabello, Luis Espejo-Antúnez

**Affiliations:** 1 Department of Medical-Surgical Therapy, Faculty of Medicine and Health Sciences, University of Extremadura, 06006 Badajoz, Spain; carlosfm@unex.es (C.F.-M.); luisea@unex.es (L.E.-A.); 2 Department of Physiotherapy, University of Seville, 41009 Seville, Spain; malbornoz@us.es

**Keywords:** spondyloarthritis, psychosocial factors, disability evaluation, pain catastrophizing

## Abstract

*Background and Objectives*: The objective of our study was to describe the biopsychosocial profile of individuals diagnosed with axial spondyloarthritis (AxSpA) and to analyze how their clinical characteristics interact with disease activity. *Materials and Methods*: An observational study was conducted, involving 28 participants diagnosed with AxSpA. We evaluated clinical outcomes (perceived pain, range of motion [RoM], pressure pain threshold [PPT], and proprioceptive acuity), psychosocial outcomes (the Pain Catastrophizing Scale [PCS], Tampa Scale of Kinesiophobia [TSK-11], and the Fear-Avoidance Beliefs Questionnaire [FABQ]), and AxSpA-specific indices (the Bath Ankylosing Spondylitis Metrology Index [BASMI], Bath Ankylosing Spondylitis Functional Index [BASFI], and Bath Ankylosing Spondylitis Disease Activity Index [BASDAI]). Data were analyzed using Spearman’s correlation coefficients and simple and multiple linear regression models. *Results*: Cervical and lumbar RoM values were reduced compared to established normative values for the general population. Significant associations were found between perceived pain, pain catastrophizing, and FABQ scores with both BASDAI and BASFI (*p* < 0.05). The interaction between perceived pain and pain catastrophizing (*p* < 0.001) accounted for 45.7% of the variance in BASDAI, while the interaction between perceived pain and FABQ (*p* < 0.001) explained 52.1% of the variance in BASDAI. *Conclusions*: The biopsychosocial profile of patients with AxSpA is characterized by moderate-intensity perceived pain and reduced cervical and lumbar mobility. The observed associations between BASDAI, pain catastrophizing, and fear-avoidance beliefs underscore the influence of psychosocial factors on disease progression.

## 1. Introduction

Axial spondyloarthritis (AxSpA) is a chronic inflammatory rheumatic musculoskeletal disease that includes both ankylosing spondylitis (AS) and non-radiographic axial spondyloarthritis (nr-AxSpA) [1,2]. Its prevalence is estimated to range between 0.5% and 1% of the global population, although it varies depending on geographic region and diagnostic methods used [1,3]. This condition primarily affects the spine and sacroiliac joints (sacroiliitis), causing pain, stiffness, and a progressive loss of mobility [1,4,5]. In addition, peripheral manifestations (arthritis, enthesitis, and dactylitis) and extra-musculoskeletal manifestations, such as acute anterior uveitis, inflammatory bowel disease (IBD), and psoriasis, are frequently observed [6]. These clinical manifestations result in physical disability due to impaired functional capacity, thereby reducing both physical and emotional well-being [3,7].

The primary goal in treating patients with AxSpA is to maximize long-term health-related quality of life (HRQoL) [8]. Recently, an update to the Assessment of SpondyloArthritis International Society (ASAS)-EULAR recommendations for the management of AxSpA was published [8]. Among these recommendations is an emphasis on individualizing treatment—both pharmacological and non-pharmacological—according to the patient’s current signs and symptoms. Previous studies have shown that these assessment tools are sensitive in detecting changes in disease progression in patients with AxSpA [8,9,10,11]; however, no studies have evaluated the interaction between these measures. In this context, the patient’s demographic characteristics (age and marital status), psychosocial factors (presence of cognitive disorders, socioeconomic status, and cultural or educational level), and psycho-emotional aspects (depression or anxiety) play a crucial role in the multidisciplinary management coordinated by the rheumatologist and must be considered when addressing subjective well-being and health-related quality of life [12]. Authors such as Wilk et al. (2023) [13] and Oskay et al. [14] have shown that central sensitization mechanisms and pain catastrophizing are associated with psychosocial factors, increasing levels of kinesiophobia, disability, and perceived pain intensity in patients with rheumatic inflammatory disorders [13,14]. Moreover, erroneous beliefs among individuals with musculoskeletal pain symptoms can perpetuate avoidance patterns and hinder the maintenance or restoration of function and social participation [15].

Despite existing studies on the relationship between psychosocial factors and perceived pain in rheumatic inflammatory disorders [8,16,17,18] and the necessary monitoring of AxSpA progression—including patient-reported outcomes in addition to clinical findings, laboratory tests, and imaging [8]—there are few studies that have investigated the interaction between these psychosocial factors and specific (BASMI, BASFI, and BASDAI) or non-specific (perceived pain, RoM, PPT, or JPS) outcome measures in patients with AxSpA. Redeker et al. [19] observed higher disease activity in patients diagnosed with AxSpA who had depressive symptoms. Similarly, these authors reported similar associations between disease-specific features and psycho-emotional aspects (e.g., anxiety) [20]. Considering that the optimal management of patients with AxSpA requires a combination of treatment modalities (e.g., drugs, education, physiotherapy, cognitive–behavioral therapy, and exercise) that address both clinical and psychosocial factors [8,13,14], the aim of this study was to describe the clinical and psychosocial factors of patients diagnosed with AxSpA and analyze their influence on disease progression.

## 2. Materials and Methods

### 2.1. Design

An observational, cross-sectional study was designed and supervised by the Bioethics Committee of the University of Extremadura (ethics approval number 51/2024). The study was prospectively registered at ClinicalTrials.gov (NCT06688929) and conducted in accordance with the Strengthening the Reporting of Observational Studies in Epidemiology (STROBE) guidelines [21]. 

### 2.2. Participants

**Figure 1:**
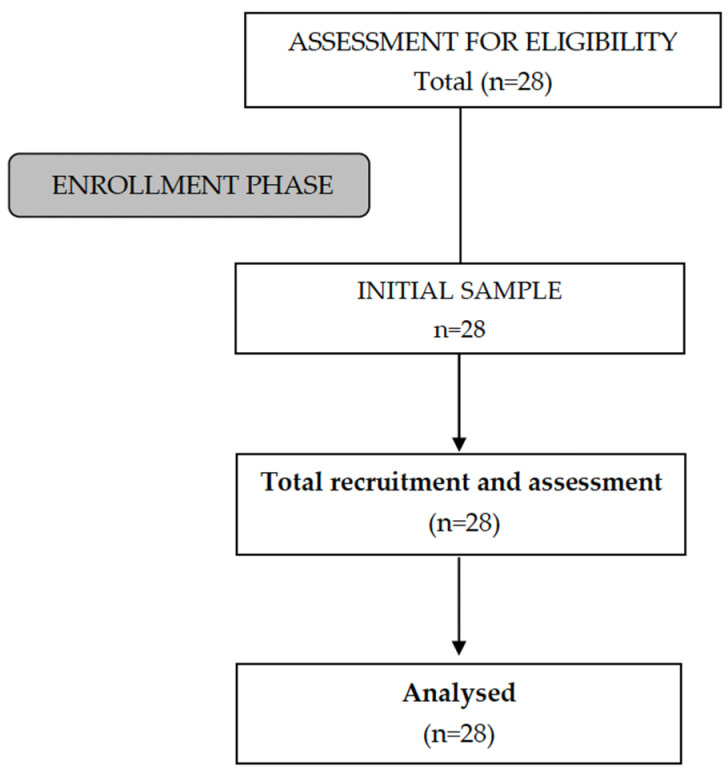
Flow diagram of the participants throughout the study.

An initial, potentially eligible sample of 34 patients with AxSpA was recruited from March to July 2024. Participants were identified through the Extremadura Association of Patients with Axial Spondyloarthritis (AEXPE, by its initials in Spanish), where a call for participation was disseminated among members using informational brochures and flyers. The participants were aged between 40 and 75 years and were in a situation of temporary (20%) or permanent disability (80%). The educational level of the sample was pre-university or technical education. The inclusion criteria were (i) adult patients (both male and female) diagnosed with AxSpA according to the Assessment of SpondyloArthritis International Society (ASAS) criteria [4]; (ii) patients who were experiencing symptoms of axial or peripheral involvement; and (iii) a minimum score of 3/10 on the Numeric Pain Rating Scale (NPRS) reported during the initial assessment. The exclusion criteria were (i) individuals with other rheumatic or musculoskeletal conditions that could affect the spine or joints (such as rheumatoid arthritis or osteoarthritis); (ii) a history of spinal surgery or joint replacement surgery; (iii) having received corticosteroid injections or other pharmacological treatments targeting inflammation within six weeks prior to data collection; and (iv) any ongoing medical–legal conflicts that could interfere with study participation. A flow diagram illustrating participant progress through the study is provided in Figure 1.

### 2.3. Assessment

The assessment of the participants was conducted at the facilities of the Faculty of Medicine and Health Sciences, University of Extremadura (Badajoz, Spain). All measurements and assessments were performed by a physical therapist with extensive experience in the evaluation and treatment of rheumatic conditions.

#### 2.3.1. Clinical Outcome Measures

- The **Numeric Pain Rating Scale (NPRS)** is an 11-point numeric rating scale, where 0 denotes “no pain” and 10 denotes “the maximum pain imaginable”. The minimum clinically important difference (MCID) for this tool was established at 1.5 points, and the minimum detectable change (MDC) was established at 2.6 points in individuals with neck pain. The NPRS is a valid scale with moderate test–retest reliability in this population (intraclass coefficient correlation (ICC): 0.76, 95% CI 0.58 to 0.93) [22].
- The **pressure pain threshold (PPT)** was assessed at specific pain points in the upper trapezius, lumbar erector spinae at L3–L4, and epicondyle muscles. The epicondyle was included as a distal reference point to serve as a control measure, helping to contrast pressure sensitivity at sites unrelated to the primary area of involvement [23]. A mechanical pressure algometer (Baseline^®^ Fabrication Enterprise, Inc., White Plains, NY, USA) with a 1 cm^2^ contact area was used. The test measures the minimum amount of pressure required for the sensation to shift from pressure to pain, at which point the participant signals “now”, and the algometer is immediately withdrawn, recording the applied pressure in kg/cm^2^ [24]. Three measurements were taken at each point with a 2 min rest interval between trials, and the mean of the three measurements was used for analysis. Previous studies have reported moderate-to-high reliability for this procedure (ICC: 0.62–0.81) and a minimal clinically detectable change (MCID) ranging from 1.1 kg/cm^2^ to 1.5 kg/cm^2^ [24].
- The cervical **range of motion (ROM)** was measured using a head goniometer (Enraf-Nonius© BV, Rotterdam, The Netherlands) to assess movements in the sagittal (flexion/extension), frontal (right/left lateral flexion), and transverse planes (right/left rotation). Each movement was performed three times with a 30 s rest period. In individuals with neck pain, the device’s standard error ranges from 2.9° (left rotation) to 4.1° (flexion), with an MDC between 5.9° and 9.6° [25]. The lumbar range of motion was assessed using the modified Schober test, marking 5 cm below and 10 cm above the lumbosacral junction in a standing position to achieve an initial 15 cm distance [26]. The patient then fully flexes forward, and the increase in distance between the marks measures lumbar flexion. This method has an ICC of 0.77 and an MDC of 1.8 cm [26].
- **Proprioceptive acuity** was assessed using two tests: the cervical joint position sense error (JPSE) and the lumbar repositioning error (LRE). The JPSE test evaluated the ability to reposition the head to its natural posture, providing a measure of cervical proprioception [27]. For this assessment, we used the Motion Guidance Clinic Kit (Motion Guidance LLC, Denver, CO, USA), which is a visual feedback device. The JPSE is a valid and reliable test used in clinical evaluation, with ICCs ranging from 0.30 to 0.78 and an MDC between 0.44° and 0.63° [28]. For the LRE assessment, participants actively flexed their lumbar spine from 0° to 30°, guided by the evaluator. After memorizing the final position for 10 s, they returned to the initial position and were then asked to reproduce the 30° lumbar flexion independently. The lumbar repositioning error (LRE) was measured using an inclinometer (iPhone^®^ smartphone app, Apple Inc., Cupertino, CA, USA) [29].

#### 2.3.2. Psychosocial Outcome Measures

- **Pain Catastrophizing Scale (PCS):** The Spanish version of the PCS is a self-administered scale (Likert scale) of 13 items and is one of the most used and reliable to assess pain catastrophizing [13,18]. Participants are asked to refer to their past painful experiences and indicate the degree to which they experienced each of the 13 thoughts or feelings: the score ranges from 0 (never) to 4 (always). The Spanish PCS version has demonstrated good validity and reliability (Cronbach’s α = 0.79; ICC = 0.84) [30].
- **Tampa Kinesiophobia Scale (TSK-11)**: The Spanish version of the TSK-11 is used to assess fear of movement and re-injury [31]. It contains 11 items, each rated on a 4-point Likert scale (1 = “strongly disagree” to 4 = “strongly agree”), with total scores ranging from 11 to 44. Higher scores indicate greater fear of pain, movement, and injury. This version has demonstrated good reliability and validity (Cronbach’s alpha: 0.79) [31].
- **Fear-Avoidance Beliefs Questionnaire (FABQ)**: This test is a self-reported measure designed to assess fear-avoidance beliefs related to physical activity and work in individuals with musculoskeletal pain. It consists of two subscales: one for physical activity (FABQ-PA) and another for work-related fear-avoidance (FABQ-W). Each item is rated on a 7-point Likert scale, with higher scores indicating stronger fear-avoidance beliefs. The Spanish version of the FABQ has demonstrated good reliability, with a Cronbach’s alpha of 0.93 and an intraclass correlation coefficient (ICC) of 0.97 [32].

#### 2.3.3. Specific Outcomes in AxSpA

- **Bath Ankylosing Spondylitis Metrology Index (BASMI):** This is a valid and reliable index designed to measure spinal mobility in individuals with ankylosing spondylitis and other spondyloarthropathies [33]. The BASMI includes five specific measurements: lateral lumbar flexion, tragus-to-wall distance, cervical rotation, lumbar flexion (measured by the modified Schober test), and intermalleolar distance [33]. Each measure is scored on a continuous linear scale from 0 to 10 based on specific formulas, with higher scores indicating greater mobility limitations [33,34].
- **Bath Ankylosing Spondylitis Disease Activity Index (BASDAI)**: This is a self-reported measure of disease activity in ankylosing spondylitis [35]. It includes six items that assess fatigue, spinal pain, joint pain and swelling, and morning stiffness, rated on a 0–10 scale [35]. The BASDAI has shown good reliability (ICC = 0.74, 95% CI: 0.52–0.88) and construct validity [36].
- **Bath Ankylosing Spondylitis Functional Index (BASFI):** This is a self-administered questionnaire used to assess physical function in patients with ankylosing spondylitis [37]. It includes 10 items that evaluate the patient’s ability to perform daily activities, rated on a 0–10 scale, with higher scores indicating greater functional limitation [37]. The BASFI has demonstrated good reliability (ICC = 0.68, 95% CI: 0.29–0.85) [36].

### 2.4. Statistical Analysis

Data analysis was performed using SPSS version 26 (SPSS Inc., Chicago, IL, USA). A descriptive analysis of sociodemographic and clinical variables was conducted, expressing continuous variables as mean ± standard deviation (SD) and median ± interquartile range (IQR). The Shapiro–Wilk test was used to assess the normality of the data. Correlation analysis was conducted to examine the relationship between relevant clinical variables using Spearman’s correlation coefficients. Subsequently, multiple linear regression analyses were performed to determine the extent to which the interaction between psychosocial variables (pain catastrophizing and fear-avoidance beliefs) and pain intensity influenced the specific AxSpA indices (BASFI and BASDAI). Additionally, to ensure the validity of the regression models used in the analysis, collinearity diagnostics were conducted by calculating the variance inflation factor (VIF), confirming that all obtained values were below 5, which is considered the threshold of problematic collinearity [21]. The level of statistical significance was set at *p* < 0.05.

### 2.5. Sample Size Estimation

Sample size calculation was conducted using G*Power 3.1 software (Düsseldorf, Germany). To detect a significant correlation with an expected effect size (ρ) of 0.55, a two-tailed test, an alpha level of 0.05, and a power of 85%, a minimum of 25 participants was required. This estimate was based on the bivariate normal model for correlation analysis. The final sample size in the study was adjusted according to this requirement.

## 3. Results

Table 1 shows the anthropometric characteristics of the participants, including age, height, weight, and body mass index (BMI). After applying the inclusion and exclusion criteria, the final sample consisted of 28 patients with AxSpA, equally distributed by gender (14 women and 14 men). A total of 81.5% of the samples were receiving pharmacological treatment, while 18.5% were not taking any drugs. The pharmacological intake was distributed as follows: 55.5% were taking more than one drug, combining disease-modifying antirheumatic drugs (DMARDs) with paracetamol/NSAIDs or corticosteroids (60%); analgesics/NSAIDs with antidepressants (26.7%); or analgesics/opioids with antidepressants (13.3%). Finally, 25.9% of the participants were taking only one oral drug, with DMARDs being the most common (71.4%), followed by antidepressants (28.6%).

Participants reported a mean perceived pain score of 6.57 ± 2.16 on the NPRS. Psychosocial variables also showed moderate scores, with a mean of 25.42 ± 11.24 out of 52 on the PCS, 46.13 ± 25.02 out of 96 on the FABQ, and 37.13 ± 10.02 out of 44 on the TSK-11.

**Figure 2:**
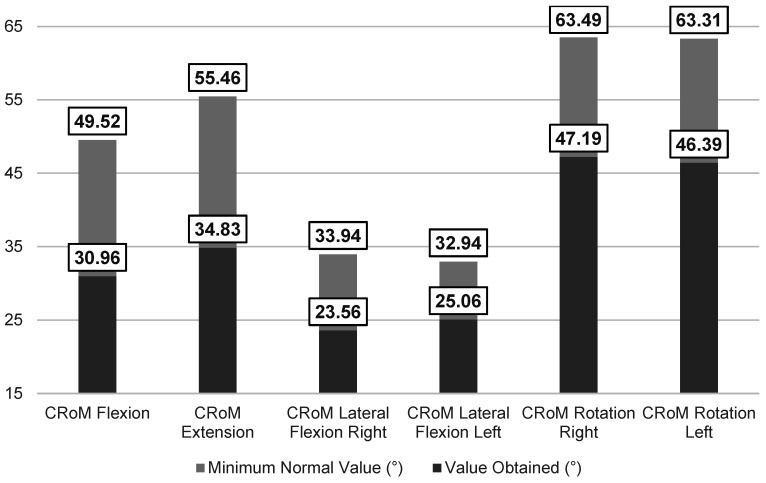
Comparative analysis of the mean values obtained for cervical RoM versus cut-off values in the neck pain population [38].

The results of the clinical outcome measures revealed limitations in all CRoM parameters, with values below those reported as normal [38] (Figure 2). Lumbar flexion (Schober test) was also reduced compared to reported cut-off points [26]. Regarding the PPT, the right trapezius muscle (1.34 ± 0.75 kg/cm^2^) and right epicondyle (1.71 ± 0.99 kg/cm^2^) exhibited greater mechanosensitivity. For cervical proprioceptive acuity (JPSE), participants showed mean values exceeding 4.5° (4.90 ± 0.77°), which is considered the minimum threshold of normality according to Revel et al. [27]. In the LRE test, participants demonstrated a mean value of 3.61 ± 3.15°, exceeding the average error range of 1–2° reported in pain-free subjects [39].

Table 2 shows the specific metrics of patients with AxSpA. The BASMI showed an average score of 4.73 ± 1.47. The greatest limitations were observed in the intermalleolar distance (29.08 ± 22.45 cm; BASMI: 8.88 ± 1.39) and cervical rotation (46.06 ± 11.28°; BASMI: 5.06 ± 1.25), followed by lateral flexion (12.84 ± 9.15 cm; BASMI: 4.49 ± 2.74) and the modified Schober test (5.18 ± 2.69 cm; BASMI: 3.58 ± 3.45). The BASDAI and BASFI indices showed mean scores of 5.87 ± 1.63 and 5.40 ± 2.34, respectively.

The correlation analysis showed significant associations between psychosocial variables and specific AxSpA indices (Table 3). BASDAI was positively correlated with NPRS (0.569, *p* < 0.01), PCS (0.622, *p* < 0.01), and FABQ (0.723, *p* < 0.01). BASFI also showed significant positive correlations with NPRS (0.376, *p* < 0.05), PCS (0.663, *p* < 0.01), and FABQ (0.719, *p* < 0.01). In the linear regression analysis, the interaction between psychosocial factors and pain intensity was a significant predictor of disease activity and functionality levels in AxSpA (Table 4). Specifically, the interaction NPRS*PCS explained 45.7% of the variance in BASDAI (*p* < 0.001), and the interaction NPRS*FABQ explained 52.1% (*p* < 0.001). For BASFI, the interaction NPRS*FABQ accounted for 45.5% of the variance (*p* < 0.001), while the interaction PCS*FABQ explained 45.2% (*p* < 0.001).

## 4. Discussion

The primary objective of this study was to describe the main clinical and psychosocial factors in patients diagnosed with AxSpA and analyze their influence on disease activity. The results obtained are consistent with previous studies highlighting the clinical relevance of psychosocial factors in disease progression [13,14]. Specifically, the fear-avoidance model of pain has been used as a framework to explain the development and persistence of symptoms following an acute episode of musculoskeletal pain [40,41]. The significant associations observed between BASDAI, pain catastrophizing, and fear-avoidance beliefs may partially account for symptom progression in patients with AxSpA.

Despite this, symptomatic management of the disease in the studied population identified a high daily consumption of drugs targeting various clinical manifestations of the disease (both specific and non-specific). Similarly, the types of drugs used were diverse, with DMARDs, NSAIDs, opioids, and antidepressants being prominent, as reported by Ramiro et al. [8]. Over the past decade, Redeker et al. [19] reported moderate-to-severe depressive symptoms in patients with AxSpA. These authors found greater depressive symptoms in those patients with higher disease activity and greater functional impairment. The fact that 40% of the population in the present study consumed antidepressants in combination with other drugs (analgesics, NSAIDs, or opioids), and 28.6% used antidepressants as their only drug, may indicate the presence of other psycho-emotional factors not analyzed in the present study that could be contributing to the perceived pain experience and its impact.

The perceived pain (NPRS), pain catastrophizing (PCS), and fear-avoidance beliefs (FABQ) were strongly associated with functionality (BASFI) and disease activity (BASDAI) (Table 3). Specifically, the regression analysis showed that the combination of these outcome measures explained a significant proportion of the variance in both indices, identifying the interaction between perceived pain and fear-avoidance beliefs as a key factor in predicting disease activity (Table 4). These variables have previously been identified as predictors of work absenteeism in patients with chronic low back pain [42]. This finding is consistent with previous studies indicating that such psychosocial factors are associated with greater disease activity [13].

No significant associations were found between the psychosocial variables analyzed (PCS, FABQ, and TSK-11) and BASMI. Similarly, kinesiophobia (TSK-11) did not show a significant correlation with disease activity (BASDAI) or functionality (BASFI) (Table 3). These results are broadly consistent with those reported by Oskay et al. [14]. It is possible that, in our sample, the influence of pain catastrophizing and erroneous beliefs about pain was more decisive than kinesiophobia, thereby reducing its impact on functionality and disease activity.

From a clinical perspective, these results suggest that the management of AxSpA should extend beyond conventional physical interventions, incorporating strategies to reduce catastrophizing and modify erroneous beliefs in order to break the fear-avoidance cycle that leads to functional decline [41]. In patients with chronic spinal pain, catastrophizing and erroneous beliefs are associated with hypervigilance and maladaptive motor responses that increase trunk stiffness [43]. This protective reaction, possibly disproportionate to the actual nociceptive threat, may contribute to the perpetuation of pain [15,44]. In this context, interventions such as Pain Neuroscience Education (PNE), aimed at modifying these beliefs, could positively influence disease activity [44].

Regarding cervical and lumbar range of motion (RoM), we found restricted mobility, with mean values below the minimum thresholds considered normal [26,38]. Our results are consistent with previous studies that have reported limitations in these parameters within this population [9,10]. However, comparisons of RoM in AxSpA may differ from other populations due to the specific effects of inflammation on the spine and sacroiliac joints [3,10]. Nevertheless, recent studies suggest that the loss of mobility in AxSpA could be attributed to both biomechanical restrictions and the influence of psychosocial factors, as addressed in this study [5].

The PPT has been associated with pain mechanisms in patients with chronic pain. Previous studies have suggested that increased pain sensitivity in AxSpA may be related to the amplification of neural signaling, resulting in a phenomenon of generalized hypersensitivity [45]. Walton et al. [46] highlighted how the PPT can be influenced by psychosocial factors, such as pain catastrophizing or kinesiophobia. In our study, we found high mechanosensitivity at all analyzed points, with values below 3 kg/cm^2^ (indicative of high sensitivity) [46]. Bodes-Pardo et al. [23] used the lateral epicondyle as a reference point to assess pain sensitivity possibly related to central sensitization in subjects with chronic low back pain and reported greater pressure pain thresholds than those observed in our sample (3.8–3.9 kg/cm^2^ vs. 1.71–1.80 kg/cm^2^). This level of sensitivity may indicate alterations in neural processing suggestive of amplification of central pain mechanisms [47].

Our results in the JPSE and LRE tests show values above the ranges considered normal in subjects without proprioceptive impairment [26,27]. This alteration in proprioception is consistent with findings reported by Mirza et al. [48] in a recent study. These authors identified a decrease in trunk position sense accuracy and an increase in postural sway in patients with AxSpA [48]. Previous studies have documented impaired proprioceptive acuity in patients with AxSpA, highlighting the influence of inflammatory factors and structural rigidity on alterations in proprioceptive receptors [11]. In this regard, interventions involving specific exercises aimed at improving proprioceptive acuity have proven effective in reducing repositioning errors in patients with AxSpA [49].

### Clinical Implications and Limitations

This study highlights the importance of a comprehensive biopsychosocial assessment in patients with AxSpA through a battery of valid and reliable tests addressing both physical and psychosocial aspects. The identified interactions suggest that the analyzed psychosocial factors could be key predictors of functionality and disease activity. This multidimensional approach provides a foundation for more personalized interventions.

On the other hand, this study has some limitations that should be considered. First, although the sample size was calculated to achieve adequate statistical power, the final number of participants is limited, which restricts the extrapolation of results to other populations with different sociocultural or environmental contexts (e.g., rural vs. urban settings). Additionally, this study did not include a comparison or control group, which limits the interpretation of results exclusively to the evaluated population. Future studies should consider the inclusion of a control/comparison group.

## 5. Conclusions

Patients with AxSpA exhibit a biopsychosocial profile characterized by limitations in cervical and lumbar range of motion and deficits in proprioceptive acuity compared to established cut-off points. Perceived pain intensity, catastrophizing, and fear-avoidance beliefs have been identified as predictive factors for functionality (BASFI) and disease progression (BASDAI). Future studies are needed to analyze the clinical effectiveness of interventions targeting a biopsychosocial approach to AxSpA.

## Figures and Tables

**Table 1:** Sociodemographic, clinimetric, and biopsychosocial characteristics of participants.

	Total Sample (*n* = 28)		
Variable	Mean (SD)	Median (IQR)	Min–Max
Age (years)	54.33 (8.76)	54.00 (15)	42–72
Height (cm)	168.63 (10.67)	173.00 (20)	149–183
Weight (kg)	74.92 (14.71)	74.50 (22)	52–102
BMI (kg/m^2^)	0.26 (0.04)	0.25 (0.06)	0.20–0.33
NPRS (0–10)	6.57 (2.16)	6.88 (2.00)	3.00–10.00
Pressure Pain Threshold (PPT)			
PPT L3–L4 Left (kg/cm^2^)	2.39 (1.14)	2.15 (1.69)	0.78–5.00
PPT L3–L4 Right (kg/cm^2^)	2.27 (0.86)	2.00 (0.86)	1.05–5.00
PPT Trapezius Left (kg/cm^2^)	1.75 (1.53)	1.37 (0.92)	0.25–5.75
PPT Trapezius Right (kg/cm^2^)	1.34 (0.75)	1.26 (0.48)	0.45–3.95
PPT Epicondyle Left (kg/cm^2^)	1.80 (0.98)	1.49 (1.03)	0.67–5.00
PPT Epicondyle Right (kg/cm^2^)	1.71 (0.99)	1.54 (0.76)	0.50–5.00
Cervical Range of Motion (CRoM)			
Flexion (°)	30.96 (14.63)	32.00 (25.5)	7.0–50.0
Extension (°)	34.83 (13.63)	32.50 (14.00)	18.00–71.00
Left Lateral Flexion (°)	25.06 (13.57)	21.75 (14.1)	8.0–55.5
Right Lateral Flexion (°)	23.56 (13.32)	22.00 (19.8)	4.0–52.5
Right Cervical Rotation (°)	47.19 (13.75)	47.00 (21.6)	18.0–68.0
Left Cervical Rotation (°)	46.39 (10.25)	46.00 (16.25)	29.00–65.00
Cervical JPSE (°)	4.90 (0.77)	4.83 (0.69)	3.00–6.00
Lumbar LRE (°)	3.61 (3.15)	2.17 (4.59)	0.33–12.00
PCS (0–52)	25.42 (11.24)	26.00 (12)	0–46
TSK-11 (11–44)	37.13 (10.02)	38.00 (15)	18–54
FABQ (0–96)	46.13 (25.02)	49.00 (50)	0–77
Modified Schober (cm)	5.18 (2.69)	6.00 (4.00)	0.00–8.70

BMI: Body mass index; FABQ: Fear-Avoidance Beliefs Questionnaire; IQR: interquartile range; JPSE: joint position sense error; LRE: lumbar repositioning error; NPRS: Numeric Pain Rating Scale; PCS: Pain Catastrophizing Scale; ROM: range of motion; SD: standard deviation; TSK-11: Tampa Scale for Kinesiophobia.

**Table 2:** Results of specific AxSpA indices.

	Total Sample (*n* = 28)		
Variable	Mean (SD)	Median (IQR)	Min–Max
BASMI linear (0–10)	4.73 (1.47)	4.46 (2.11)	2.41–7.96
Lateral Flexion (cm)	12.84 (9.15)	11.00 (9.74)	2.63–47.38
(0–10)	4.49 (2.74)	4.45 (3.86)	0.00–9.29
Tragion-to-Wall Distance (cm)	13.69 (4.79)	12.00 (1.4)	8.75–27.00
(0–10)	1.81 (1.49)	1.33 (0.35)	0.25–6.33
Modified Schober (cm)	5.18 (2.69)	6.00 (4.00)	0.00–8.70
(0–10)	3.58 (3.45)	2.57 (5.97)	0.00–10.00
Intermalleolar Distance (cm)	29.08 (22.45)	32.00 (47.10)	0.90–64.50
(0–10)	8.88 (1.39)	9.77 (2.53)	6.00–10.00
Cervical Rotation (°)	46.06 (11.28)	43.00 (22.22)	29.0–65.0
(0–10)	5.06 (1.25)	5.39 (2.39)	2.86–7.09
BASDAI (0–10)	5.87 (1.63)	6 (1.7)	0.4–8.3
BASFI (0–10)	5.40 (2.34)	5.20 (3.8)	0.0–8.9

BASMI: Bath Ankylosing Spondylitis Metrology Index; BASDAI: Bath Ankylosing Spondylitis Disease Activity Index; BASFI: Bath Ankylosing Spondylitis Functional Index.

**Table 3:** Correlation coefficients between psychosocial outcomes and specific AxSpA indices.

	Total Sample (*n* = 28)			
	NPRS	PCS	FABQ	TSK-11
BASMI	0.058	−0.011	−0.002	0.067
BASDAI	0.569 **	0.622 **	0.723 **	−0.100
BASFI	0.376 *	0.663 **	0.719 **	−0.171

BASDAI: Bath Ankylosing Spondylitis Disease Activity Index; BASFI: Bath Ankylosing Spondylitis Functional Index; BASMI: Bath Ankylosing Spondylitis Metrology Index; FABQ: Fear-Avoidance Beliefs Questionnaire; NPRS: Numeric Pain Rating Scale; PCS: Pain Catastrophizing Scale; TSK-11: Tampa Scale for Kinesiophobia. Spearman’s correlation analysis: * indicates a significant correlation at the 0.05 level, and ** indicates a significant correlation at the 0.01 level.

**Table 4:** Linear regression analysis of the association between psychosocial factor interactions and specific AxSpA indices (BASDAI and BASFI).

	Total Sample (*n* = 28)			
	R^2^	β	*t*	*p*-Value
BASDAI				
NPRS × PCS	0.457	0.676	4.675	<0.001
NPRS × FABQ	0.521	0.722	5.315	<0.001
PCS × FABQ	0.381	0.618	4.003	<0.001
BASFI				
NPRS × PCS	0.405	0.636	4.205	<0.001
NPRS × FABQ	0.455	0.674	4.657	<0.001
PCS × FABQ	0.452	0.672	4.631	<0.001

BASDAI: Bath Ankylosing Spondylitis Disease Activity Index; BASFI: Bath Ankylosing Spondylitis Functional Index; BASMI: Bath Ankylosing Spondylitis Metrology Index; FABQ: Fear-Avoidance Beliefs Questionnaire; NPRS: Numeric Pain Rating Scale; PCS: Pain Catastrophizing Scale.

## Data Availability

The data presented in this study are available upon request from the corresponding author.
